# Evaluating Longitudinal Community Health Worker–Led Interventions for Improving Hypertension Management in Urban U.S. Communities: A Rapid Review

**DOI:** 10.1016/j.focus.2026.100527

**Published:** 2026-09-10

**Authors:** Sagar Rastogi, Nisa Maruthur, Maxwell Droznin

**Affiliations:** 1 Johns Hopkins University, Baltimore, Maryland; 2 Department of Medicine, Johns Hopkins University School of Medicine, Baltimore, Maryland

**Keywords:** Community health workers, hypertension, urban health, prevention, rapid review, blood pressure

## Abstract

**Introduction:**

Underserved urban U.S. communities experience a disproportionate burden of uncontrolled hypertension, driven by structural and socioeconomic barriers. Community health workers may support sustained improvements in hypertension control and self-management.

**Methods:**

The authors systematically searched PubMed (2015–2025) for U.S.-based, urban, longitudinal community health worker–led interventions recruiting participants directly from community settings with reported pre/post blood pressure outcomes. Studies conducted in rural settings, recruiting from clinical settings, or lacking pre/post blood pressure data were excluded. Two investigators independently screened, extracted data, and assessed the risk of bias. Heterogeneity precluded meta-analysis; findings were synthesized narratively with attention to the evidence hierarchy.

**Results:**

Ten studies met the inclusion criteria: 3 RCTs, 1 non-RCT, and 6 pre–post designs. Across the studies, community health worker–led programs commonly reduced blood pressure and improved self-management behaviors. Six studies (60%) reported significant reductions in blood pressure (systolic blood pressure: 2.3–20 mmHg; diastolic blood pressure: 1.6–7.4 mmHg). However, only 1 of the 3 RCTs demonstrated significant between-group differences in blood pressure; 2 showed no significant effect versus the controls. Interventions lasting at least 6 months consistently demonstrated significant improvements in blood pressure, whereas 3-month programs were less likely to show a significant change. Several studies reported improved medication adherence and increased self-monitoring of blood pressure at home.

**Discussion:**

Longitudinal, community-based community health worker–led interventions are associated with improved hypertension control and self-management in underserved urban U.S. communities. Multicomponent, culturally tailored programs of at least 6 months that integrate medication support and linkage to care appear to be most effective. Future research should prioritize larger, rigorously designed RCTs, cost-effectiveness, and implementation strategies to enable the widespread implementation of effective community health worker models.

## Highlights

• Community health worker (CHW) programs of ≥6 months were associated with blood pressure (BP) reductions.
• CHW programs of 3 months were rarely associated with BP drops.
• Largest improvements were seen among participants with uncontrolled BP at baseline.
• Hybrid group classes plus 1:1 support showed promise over single-mode delivery.
• CHW–pharmacist/dietitian collaborations were associated with favorable outcomes.

## Graphical Abstract

**Figure:**
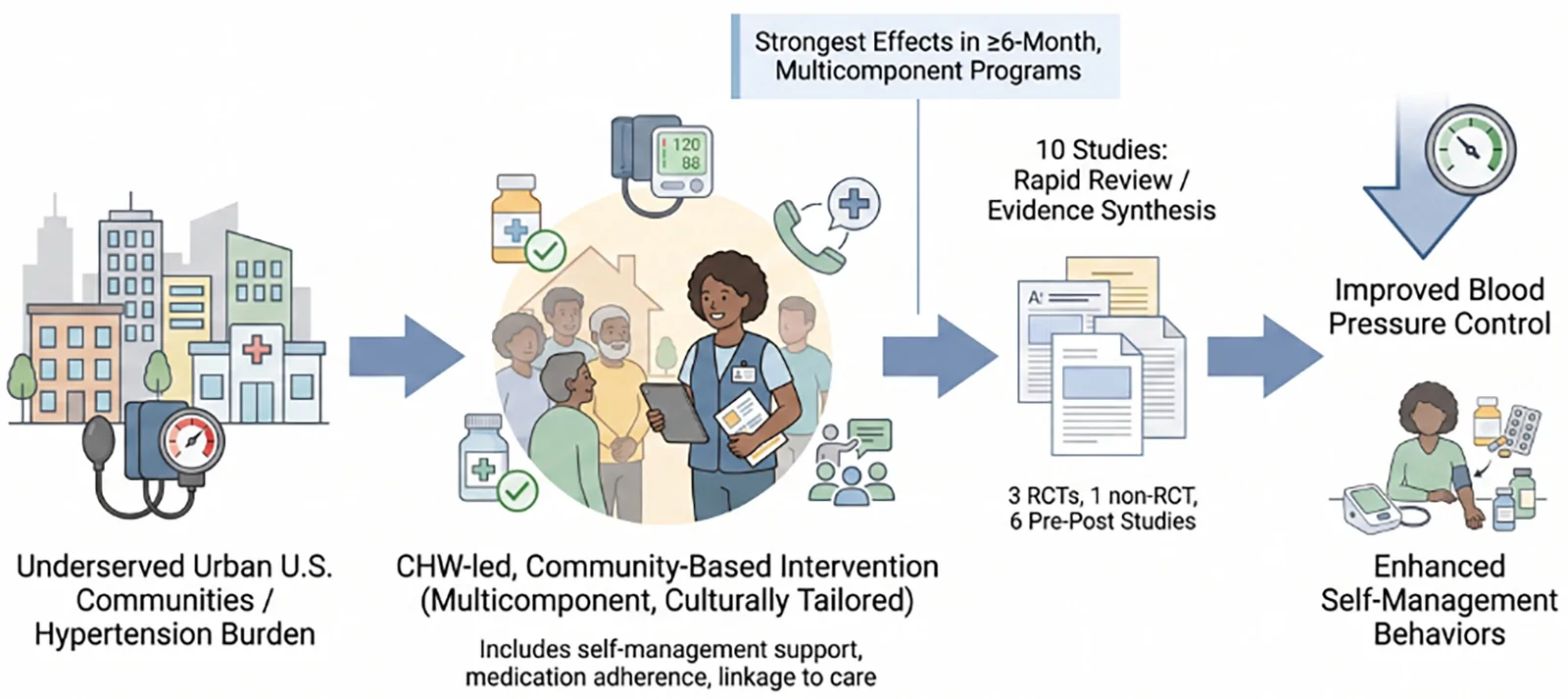

## INTRODUCTION

Approximately 45% of the U.S. adult population is affected by hypertension,^1^ elevating the risk of heart attack, stroke, and kidney disease among many other chronic and acute health complications, including sudden death, leading to significant health burdens among individuals, their families, and the healthcare system as a whole. In 2020 alone, the economic toll of uncontrolled hypertension reached over $51 billion in the U.S.^2^ Risk factors for hypertension include low SES, a high-sodium diet, physical inactivity, obesity, and alcohol/drug use. Significant racial and socioeconomic health disparities exist in the identification and treatment of hypertension, with non-Hispanic Black Americans in underserved urban communities shouldering the highest burden of uncontrolled hypertension than other demographics.^3^ Early identification and treatment of hypertension are critical to mitigating the long-term adverse health consequences of the disease.

Multiple well-established barriers compound the disproportionate prevalence of the hypertension burden placed on vulnerable low socioeconomic urban populations.^3^^,^^4^ For example, structural barriers to accessing primary care frequently delay diagnosis, and concerns about medication costs lead to suboptimal adherence to treatment plans.^5^ Knowledge gaps related to uncontrolled hypertension are linked to reduced awareness of risk factors and the serious health consequences that result from noncompliance and ineffective management.^5^ The asymptomatic nature of hypertension also serves as a notable barrier to blood pressure control, because many individuals delay urgent treatment until obvious complications manifest.^6^ In the same way, medication adherence is negatively affected by the patient perception that prescribed medications are ineffective in controlling their hypertension.^6^ In addition, the increased prevalence of comorbidities such as obesity and Type 2 diabetes among these underserved socioeconomic groups further exacerbates the prevalence and severity of their uncontrolled hypertension.^7^^,^^8^ A combination of the risks and burdens associated with receiving medical care for hypertension management creates a cycle of late diagnosis and insufficient treatment, potentially explaining the higher rates of cardiovascular morbidity observed in underserved non-Hispanic Black and Hispanic populations.^7^, ^8^, ^9^

Community health workers (CHWs) are frontline public health workers who have been widely deployed since the 1970s in low- and middle-income countries to address gaps in health care.^10^ Particularly, CHWs have been effective in providing tailored health education; conducting basic health screenings; and facilitating the linkage of individuals to formal medical care, which is often lacking in many developing countries.^11^ In the U.S., there is an increasing presence of CHWs being recognized as a valuable resource in helping patients with adherence to treatment and self-management of chronic conditions such as diabetes, asthma, and hypertension.^12^ As the role of CHWs continues to evolve within the U.S., there is strong evidence that suggests that they are effective in reducing health disparities and improving the health and well-being of the communities they serve.^13^

The evidence base for CHW involvement in hypertension management has emerged over the past decade. Several recent meta-analyses have examined CHW-led interventions for blood pressure management. Mills et al.^14^ found that pharmacist-led and CHW-led interventions significantly outperform physician-led interventions, whereas Patil and colleagues^15^ concluded that lay advisor interventions improve hypertension outcomes but highlighted the need to better understand successful implementation factors. In addition, the Community Preventive Services Task Force completed a comprehensive review of Community Health Workers in Hypertension in 2015, finding sufficient evidence to recommend CHW interventions for improving blood pressure outcomes, particularly among underserved populations.^16^ These reviews collectively suggest that CHWs can contribute to hypertension management, but questions remain regarding which intervention characteristics, populations, and implementation contexts are most effective. This review builds upon these findings by specifically examining the PubMed database, which was not systematically searched in these prior analyses, and by focusing exclusively on community-recruited populations in urban U.S. settings.

## METHODS

A CHW is defined by the NIH as a trained individual who contributes to disease prevention by educating community members, encouraging healthy behaviors, and providing social services to facilitate their overcoming healthcare disparities.^17^ Studies using alternative terminology (e.g., lay health advisors, promotores de salud, community health advisors) were included only if the described role met this operational definition. Longitudinal CHW support was defined as interventions involving repeated CHW contact over a minimum of 3 months. An urban setting was defined according to the U.S. Census Bureau classifications or as explicitly stated by study authors.

This rapid review follows streamlined systematic review methods, using a single database search strategy to provide timely evidence synthesis while maintaining methodologic rigor in study selection, data extraction, and quality assessment. The search included studies published in the PubMed database between 2015 and 2025 to provide an analysis of the most recent literature relevant to hypertension management.^18^ The complete search strategy, including all MeSH (Medical Subject Headings) terms and filters, is provided in Appendix 1 (available online). Studies were included if they were conducted with humans in an urban area in the U.S., were published in a peer-reviewed scientific journal, were written in English, included longitudinal CHW support, reported preintervention and postintervention blood pressure measurements, and recruited participants from community-based settings. Studies were included if blood pressure was designated as either a primary or secondary outcome and CHWs provided education on hypertension management. The authors acknowledge that interventions with blood pressure as a primary versus secondary outcome may differ in their intensity and focus; therefore, results are stratified by this distinction where appropriate. The authors chose to exclude studies recruiting participants from clinical settings to focus on the effectiveness of interventions reaching individuals who may be disconnected from the health system. This review was conducted and reported in accordance with PRISMA 2020 guidelines (Appendix 2, available online, provides the completed PRISMA checklist).

Two investigators, SR and MD, independently identified and assessed studies from the PubMed search results. After establishing the inclusion criteria, the studies were sequentially evaluated through title review, abstract review, and full-text review. Each study was advanced to the next phase of review only upon agreement by both investigators. Disagreements regarding inclusion or whether a study should be advanced to the next phase of review were resolved through discussion. The reference lists of included studies were reviewed to identify additional potentially eligible studies not captured by the electronic search.

Once the final studies were selected, both investigators (SR and MD) independently performed data abstraction and quality assessment. Nonrandomized studies were evaluated for bias using the Joanna Briggs Institute checklist for quasi-experimental studies, whereas RCTs were evaluated using the original Cochrane Risk of Bias tool. Disagreements regarding study quality were resolved through discussion between the investigators.

Given the heterogeneity in study designs, the authors adopted a narrative synthesis approach stratified by level of evidence. RCTs were considered to be the highest level of evidence for efficacy, followed by quasi-experimental studies with comparison groups and finally pre–post designs without controls. Findings are presented with explicit acknowledgment of study design limitations, and conclusions are weighted toward RCT findings where available. This rapid review analyzed data from previously published studies and did not involve collecting new data from human participants. Therefore, IRB approval was not required.

## RESULTS

**Figure 1:**
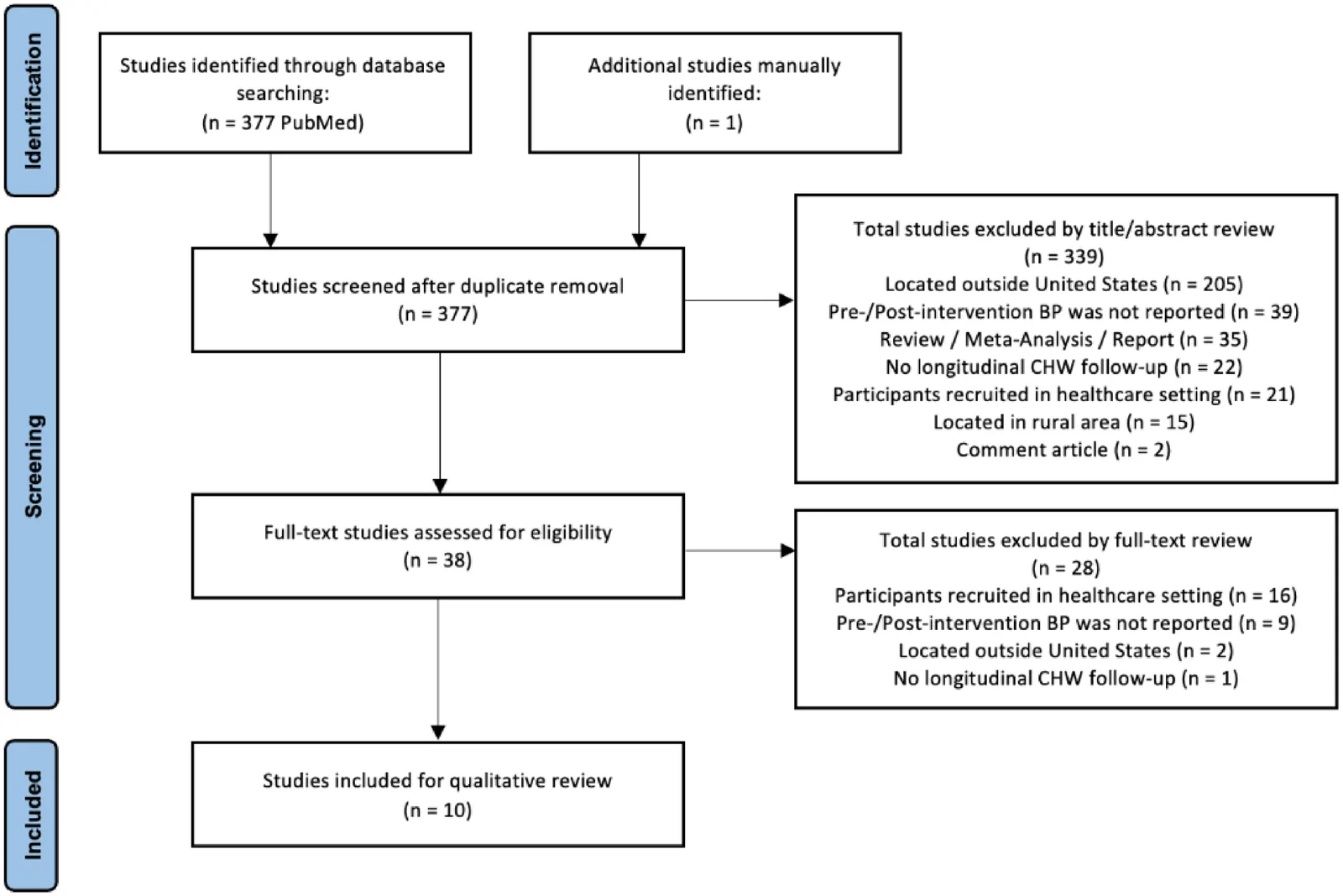
PRISMA flow diagram of study selection.

A total of 377 studies were initially identified from the PubMed search (Figure 1). All potentially eligible studies were evaluated, and 368 of them were excluded in a systematic manner. Specifically, 261 were excluded by title review, 79 were excluded by abstract review, and 28 were excluded by full-text review.

The 368 excluded publications did not meet this review’s criteria for the following reasons: 207 were located outside of the U.S.; 48 failed to report preintervention and postintervention blood pressure outcomes; 37 recruited study participants from healthcare or clinical settings; 35 were systematic reviews, meta-analyses, or report articles; 23 did not involve longitudinal support from CHWs; 15 were located in rural areas, 2 were designated by PubMed as comment articles, and 1 search result was a duplicated publication. One additional study that met all inclusion criteria and none of the exclusion criteria was manually added to the rapid review through prior knowledge in the topic area.

A total of 10 studies, published between 2017 and 2024, met the inclusion criteria for systematic review and are described in Table 1, Table 2.^19^, ^20^, ^21^, ^22^, ^23^, ^24^, ^25^, ^26^, ^27^, ^28^ The intervention durations ranged from 3 to 24 months; they were conducted in medically underserved urban communities across various regions in the U.S., including North Florida (*n*=1),^19^ New York City (*n*=2),^20^^,^^21^ Chicago (*n*=2),^22^^,^^23^ Detroit (*n*=1),^24^ Denver (*n*=1),^25^ Baltimore-Washington (*n*=1),^26^ Winston-Salem (*n*=1),^27^ and Hawaii and Washington states (*n*=1).^28^ Recruitment of participants occurred from urban housing developments, community health centers, churches, and culturally affiliated community organizations and targeted predominantly minority populations, including African American, Korean American, Filipino American, Native American, Marshallese, and Latino adults.^19^^,^^28^

**Table 1:** Included Study Characteristics

Author, year	Location	Study design	Data sources	Number of participants analyzed	Relevant demographics
Wheat,^19^ 2020	North Florida	Pre–post	Recruited through CHW networks from medically underserved areas in North Florida	33 participants with Stage 1 hypertension (SBP ≥130 mmHg or DBP ≥80 mmHg)	75.8% were Black, 18.2% were Native American, and 6.1% were Caucasian
Lopez et al.,^20^ 2017	East Harlem, New York City, New York	Non-RCT	Recruited from 5 low-income public housing developments through telephone survey and outreach at local health fairs	370 total participants (199 assigned to intervention group and 171 assigned to comparison group)	Intervention: 51.8% were Hispanic, 47.2% were non-Hispanic Black, and 1% were non-Hispanic White Comparison: 57.1% were Hispanic, 42.3% were non-Hispanic Black, and 0.6% were non-Hispanic White
Ursua et al,.^21^ 2018	New York City, New York	RCT	Recruited from traditional and nontraditional venues frequented by Filipinos in New York City	240 total participants (112 assigned to treatment group and 128 assigned to control group)	100% were Filipino-American in treatment and control groups
Lynch et al.,^22^ 2024	Chicago, Illinois	Pre–post	Recruited from churches in low-income neighborhood in Chicago	75 participants with poorly controlled blood pressure	100% were African American
Vargas et al.,^23^ 2024	Chicago, Illinois	RCT	Recruited through flyers, community events, and referrals from 3 partnering Latinx communities	40 total participants (20 assigned to PRIME2 intervention and 20 assigned to usual care)	100% were Latino; 95% were female in PRIME2 intervention and usual-care groups
Gleason-Comstock et al.,^24^ 2023	Detroit, Michigan	Pre–post	Recruited from 27 ZIP codes in Detroit through community-based organizations, churches, and home visits	96 participants	96% were African American
Krantz et al.,^25^ 2017	Denver, Colorado	Pre–post	Recruited from churches, rec centers, and senior living centers	768 participants	97% were Latino, and 3% were non-Latino
Kim et al.,^26^ 2019	Baltimore-Washington, Maryland	Pre–post	Recruited through advertisements through ethnic newspapers, social media outlets, and emails in the Baltimore–Washington area	215 participants with prediabetes or diabetes	100% were Korean American
Pedley et al.,^27^ 2018	Winston-Salem, North Carolina	RCT	Recruited through mass mailing, in-person presentation at community organizations, health fair, and workplace screening	301 total participants (151 assigned to group-based lifestyle intervention and 150 assigned to enhanced usual care)	Lifestyle Intervention: 74% were White, and 26% were non-White Usual care: 73% were White, and 27% were non-White
McElfish et al.,^28^ 2024	Hawaii and Washington	Pre–post	Recruited from churches	185 participants	100% were Marshallese Pacific Islanders

CHW, community health worker; DBP, diastolic blood pressure; SBP, systolic blood pressure.

**Table 2:** Results of Included Interventions

Author, year	Study protocol	CHW role/training	Pre–post BP	Other study outcomes
Wheat,^19^ 2020	CHW collected patient history and medication adherence barriers Collaboration with pharmacist to identify action plan Patient connected with resources to meet goals Action plan iterated at follow-ups	Trained in medication therapy management Conducted patient interviews and linked to care Provided up to 4 follow-ups over 6 month period and tracked barriers	6-month follow-up: *SBP: 136 -> 130.1= −5.9 mmHg *DBP: 85.7 -> 81.2= −4.5 mmHg	Significant improvement in medication adherence, with 75.6% of barriers being resolved by final follow-up
Lopez et al.,^20^ 2017	Intervention group: 6 sessions with CHW where BP is measured 3 times and averaged, and participant self-reported physical activity, general mental health status, self-perceived chronic disease management, healthcare access, self-efficacy, and quality of life. Comparison group: At baseline and 3-month follow up, a brief in-person questionnaire and biometric assessment of blood pressure, height, and weight were administered.	Recruited from target housing residencies Trained in core competencies and linking to care Provided intervention group with education, instrument support, goal setting at 6 sessions, and referrals to access care in Harlem	No significant change at 3-month follow-up	Significant increase in participants reporting that they are managing hypertension well (80.5 -> 88.8%) Significant increase in participants with baseline hypertension who self-monitor blood pressure (45.6 -> 60.7%) Significant increase in average number of days of physical activity (4.6 -> 6.6) 90% report satisfaction with CHW
Ursua et al.,^21^ 2018	Treatment group: Participants received 4 educational workshops and 4 one-on-one visits with CHWs over a 4-month period Control group: Participants received 1 educational workshop	CHWs were Filipino immigrants Completed 60-hour competency training Provided 1:1 individual goal-setting plans with participants, facilitated access to primary care physician, provided social support, and assured adherence to physician appointments	Treatment group at 8 months: *SBP: 145.1 -> 125.1= −20 mmHg *DBP: 84.5 -> 77.1= −7.4 mmHg Control group at 8 months: *SBP: 142.7 -> 138.4= −4.3 mm Hg DBP: no significant change	Treatment group showed significantly higher improvement in SBP At 8 months, blood pressure was controlled among a significantly greater percentage of individuals in the treatment group (83.3%) than in the control group (42.7%) Treatment group saw a significant improvement in appointment keeping with physicians
Lynch et al.,^22^ 2024	Average of 7.5 CHW visits conducted over 6 months Created action plan Reviewed BP log and any high/low readings Engaged in diet activities Reviewed progress on previous action plans	3 CHWs selected through interview process Received 50 hours of in-person training and 20 hours online Assisted participants with medication adherence, diet change, and referral to care	6-month follow-up: All participants (*n*=75): *SBP: −5.0 mmHg *DBP: −3.6 mmHg Participants with uncontrolled BP (*n*=42): *SBP: −9.2 mmHg *DBP: −5.4 mmHg ∼47.7% achieved 10-point systolic drop	Significant increase in medication adherence Intervention fidelity was poor because CHWs did not strictly follow protocol
Vargas et al.,^23^ 2024	PRIME2 curriculum consisted of an integrated group-based diabetes prevention intervention delivered by promotoras 16 group weekly sessions	Investigators reviewed session materials with bilingual promotoras (CHW) Promotoras delivered curriculum with cultural humility and knowledge of local resources when providing health education	No significant change at 3- or 6-month follow-up	PRIME2 participants attended 10.1 of 16 sessions on average
Gleason-Comstock et al.,^24^ 2023	CHW completed baseline assessment (heart health knowledge, perceived CV risk, health literacy) Nurse completed clinical assessment (weight, BP) Nurse and CHW created action plan to lower risk	CHWs were African American and had 6–23 years of outreach experience Received on-site heart health training CHW provided heart health education and developed action plan with nurse to lower CV risk	No significant change at 6-month follow-up	Significant increase in heart health knowledge Significant decrease in participant perception of personal risk for heart disease, heart attack, stroke
Krantz et al.,^25^ 2017	CHWs delivered a 12-week bilingual curriculum on nutrition, physical activity, diabetes, and risk factor awareness	CHWs were bilingual Latinos Provided participants with education, linked to health insurance enrollment, and encouraged lifestyle change and clinical follow-up	3-month follow-up: All participants (*n*=768): *SBP: 132 -> 129= −3 mmHg At-risk participants with SBP ≥140 mmHg (n=244): *SBP: 147 -> 139= −8 mmHg	Program completion (8 of 12 sessions) was associated with magnitude of reduction in SBP
Kim et al.,^26^ 2019	Goal setting Skills training included online modules and CHW met with participant 3 times to reinforce diet, meeting personal goals, using instruments, and managing stress Motivation-based reinforcement with daily messages to motivate self-management	CHWs were bilingual Used web app system to manage recruitment, enrollment, scheduling, monitoring, communication, case management, counseling, and reporting	3-month follow-up: *SBP: 128.7 -> 122.4= −6.3 mmHg *DBP: 78.6 -> 72.6= −6.0 mmHg 6 -follow-up: *SBP: 128.7 -> 124.8= −3.9 mmHg *DBP: 78.6 -> 74= −4.6 mmHg	Proportion of participants with blood pressure controlled (SBP/DBP <130/80) significantly increased by 16.3% and 11.2% at 3 and 6 months, respectively
Pedley et al.,^27^ 2018	Phase 1 (1–6 months): weekly group sessions with a goal of weight loss by decreasing caloric intake and moderate intensity exercise Phase 2 (7–24 months): monthly group session and phone call with CHW with a goal of weight maintenance	CHW trained, monitored, and supervised by registered dieticians CHW delivered curriculum; led group sessions at rec center; and connected with individual participants by phone, monthly, during phase 2	1 year follow-up: *SBP: 127.1 -> 123.1= −4 mmHg *DBP: 73.2 -> 70.5= −2.7 mmHg 2 year follow-up: *SBP: 127.1 -> 124.8= −2.3 mmHg *DBP: 73.2 -> 71.6= −1.6 mmHg	87% of the participants completed the 2-year study period
McElfish et al.,^28^ 2024	CHW delivered 10 hours of Family-DSMES education conducted in 8 sessions over 8–10-week period Curriculum covered healthy eating, being active, glucose monitoring, taking medications, problem solving, reducing risks, healthy coping, and goal setting Curriculum considered cultural norms and practices related to health behaviors that are specific to Marshallese Pacific Islanders	CHWs were bilingual in Marshallese/English Provided group education sessions in churches and encouraged goal setting	No significant change at 12-week follow-up	Significant decrease in Hba1c (this was the primary outcome studied)

BP, blood pressure; CHW, community health worker; CV, cardiovascular; DBP, diastolic blood pressure; DSMES, Diabetes Self-Management Education and Support; SBP, systolic blood pressure.

*The asterix is defined as indicating significance (pre-post BP).

Five studies employed CHWs to directly aid in the reduction of hypertension prevalence or risk of cardiovascular disease.^19^^,^^21^^,^^22^^,^^24^^,^^25^ The remaining 5 studies employed CHWs to decrease chronic disease burden or prevalence of metabolic syndrome or improve diabetes management among participants, with achieving blood pressure control as an additional measured outcome to evaluate the effectiveness of the intervention.^20^^,^^23^^,^^26^^,^^27^^,^^28^

Although all studies involved CHWs as essential components of the specified intervention, their roles varied among protocols. CHWs were responsible for recruiting participants, delivering personalized and/or group-based heart health education, counseling on the use of self-monitoring blood pressure instruments, creating goals and long-term action plans, conducting interviews to identify structural barriers to medical care, referring participants to primary care, and completing regular follow-ups to assess study adherence and changes in outcome measurements.^19^, ^20^, ^21^, ^22^, ^23^, ^24^, ^25^, ^26^, ^27^, ^28^ In 3 studies, CHWs functioned as part of a multidisciplinary team alongside pharmacists, nurses, or registered dieticians, who helped participants meet specific intervention goals by improving adherence to antihypertensive medications, tailoring action plans on the basis of clinical assessments, or enhancing diet management, respectively.^19^^,^^24^^,^^27^ Five studies utilized personalized CHW support,^19^^,^^20^^,^^22^^,^^24^^,^^26^ 3 studies used CHWs to deliver a weekly curriculum-based group education,^23^^,^^25^^,^^28^ and 2 employed a combined approach of group workshops with one-on-one meetings.^21^^,^^27^ One intervention used a digital approach where CHWs motivated participants to self-manage their health through a mobile app system, which they used for teaching lessons, monitoring measured data, and setting participant goals.^26^

CHWs were typically recruited from within the communities they served and shared linguistic, cultural, or ethnic identities with participants to limit language barriers, build trust, and improve intervention engagement.^21^^,^^23^^,^^25^^,^^26^^,^^28^ Training and selection of CHWs differed depending on the needs of each study, ranging from rigorous interview procedures based on prior experience to receiving 70 hours of in-person and online competency education covering content on cardiovascular health and behavioral coaching.^19^^,^^28^

Six of the 10 studies reported statistically significant improvements in systolic blood pressure (SBP) and/or diastolic blood pressure (DBP) from baseline measurement to final follow-up after the intervention was completed.^19^^,^^21^^,^^22^^,^^25^^,^^26^^,^^27^ SBP reductions ranged from 2.3 to 20 mmHg, whereas DBP reductions ranged from 1.6 to 7.4 mmHg.^21^^,^^27^ Two of the 3 collaborative studies involving CHWs and another specialized healthcare provider led to significantly improved blood pressure control. Specifically, the CHW–pharmacist^19^ and CHW–dietician^27^ interventions were effective in decreasing SBP and DBP, whereas the CHW–nurse^24^ team did not lead to a change at follow-up. In addition, only 33% of the studies that involved solely group-based interactions with CHWs demonstrated a significant improvement in postintervention blood pressure management among their participants.^23^^,^^25^^,^^28^ On the contrary, 60% of the studies involving solely personalized interaction between CHWs and participants^19^^,^^20^^,^^22^^,^^24^^,^^26^ and 100% of studies utilizing a combined approach of group-based workshops with additional personalized sessions led to a significant reduction in blood pressure.^21^^,^^27^ A total of 80% of studies focused on using CHWs to manage participant hypertension and decrease future risk of cardiovascular disease as the primary outcome were successful at lowering SBP and DBP. Of the remaining studies that measured hypertension as a secondary outcome in their participants but focused their CHW interventions on improving glycemic control and reducing the prevalence of metabolic syndrome, 60% failed to achieve hypertension management.

Two studies, Lynch et al.^22^ and Krantz and colleagues,^25^ were unique in identifying, separating, and analyzing data on participants with uncontrolled blood pressure (SBP ≥140 mmHg or SBP ≥130 mmHg with age ≥65 years or self-reported risk factors of cardiovascular disease, heart failure, or diabetes) or participants at risk (SBP ≥140 mmHg but without other known medical risk factors) on the basis of baseline measurements. In both studies, there was a significant change in blood pressure, with Lynch et al.^22^ reporting a 9.2 mmHg drop in SBP and a 5.4 mmHg drop in DBP, whereas Krantz and colleagues^25^ recorded an 8 mmHg drop in SBP. In addition, 47.7% of participants in Lynch et al.^22^ achieved at least a 10 mmHg drop in SBP by the end of the study.

Two of the 4 studies that failed to show a significant change in blood pressure had a shorter overall intervention duration of 3 months.^20^^,^^23^^,^^24^^,^^28^ All studies with an intervention duration lasting more than 6 months resulted in significantly decreased blood pressure.^21^^,^^27^

Four studies documented significant improvements in hypertension self-management behaviors.^19^^,^^20^^,^^21^^,^^22^ For example, Wheat and colleagues^19^ and Lynch et al.^22^ reported a significant increase in medication adherence. Specifically, Wheat and colleagues^19^ found that the CHW–pharmacist team resolved 75.6% of barriers related to antihypertensive adherence, including forgetfulness, knowledge gaps, and medication concerns. Despite no significant change in postintervention SBP or DBP, Lopez et al.^20^ observed an increase from 45.6% to 60.7% of participants with hypertension self-reporting that they routinely self-monitored blood pressure as well as all intervention participants reporting that they increased the average number of days that they participated in physical activity from 4.6 to 6.6 after 6 sessions with a CHW over a 3-month period. Ursua and colleagues^21^ were notable not only in reporting the largest drops in SBP and DBP among their study participants but also in revealing a significant improvement in appointment-keeping among their treatment group, which received 4 group-based educational workshops combined with 4 one-on-one CHW visits.

Seven of the 10 studies reviewed were nonrandomized studies, which were evaluated for study quality using the Joanna Briggs Institute checklist for quasi-experimental studies.^19^^,^^20^^,^^22^^,^^24^, ^25^, ^26^^,^^28^ Three of the 10 studies reviewed were RCTs, which were evaluated for study quality using the Cochrane Risk of Bias tool.^21^^,^^23^^,^^27^ Overall, the methodologic quality was moderate across studies, as described in Tables 3 and 4.^18^, ^19^, ^20^, ^21^, ^22^, ^23^, ^24^, ^25^, ^26^, ^27^ Common strengths included clearly defined interventions, appropriate temporal sequencing of outcomes, and repeated follow-up measurements. Six of the 7 nonrandomized studies had participants serve as their own controls,^19^^,^^22^^,^^24^, ^25^, ^26^^,^^28^ with Lopez et al.^20^ being the only study to establish separate intervention and comparison groups using nonrandom assignment. Additional sources of bias included incompletely analyzed follow-up due to study attrition, reliance on self-reported behavior outcomes, and limited reporting of statistical approaches for handling missing or confounding data.

**Table 3:** Nonrandomized Study Quality Assessment Using JBI Checklist for Quasi-Experimental Studies

Author, year	Q1	Q2	Q3	Q4	Q5	Q6	Q7	Q8	Q9	Risk of bias
Wheat,^19^ 2020	Y	N	Y	N	Y	Y	Y	Y	Y	Moderate
Lopez et al.,^20^ 2017	Y	Y	Y	N	Y	Y	Y	Y	Y	Moderate
Lynch et al.,^22^ 2024	Y	N	Y	N	Y	Y	Y	Y	Y	Moderate
Gleason-Comstock et al.,^24^ 2023	Y	N	Y	N	Y	Y	Y	Y	Y	Moderate
Krantz et al.,^25^ 2017	Y	N	Y	N	Y	Y	Y	Y	Y	Moderate
Kim et al.,^26^ 2019	Y	N	Y	N	Y	Y	Y	Y	Y	Moderate
McElfish et al.,^28^ 2024	Y	N	Y	N	Y	Y	Y	Y	Y	Moderate

Q1: Clear cause and effect are established; Q2: Participants in comparisons are similar; Q3: Participants receive similar treatment other than the intervention; Q4: A control group is present; Q5: Multiple measurements are taken before and after intervention; Q6: Follow-up is complete; Q7: Outcomes are measured in the same way; Q8: Outcomes are measured reliably; and Q9: Appropriate statistical analysis is used.

JBI, Joanna Briggs Institute; N, no; Y, yes.

**Table 4:** RCT Quality Assessment Using Cochrane Risk of Bias 2 Tool

Author, year	D1	D2	D3	D4	D5	Overall risk of bias
Ursua et al.,^21^ 2018	Low	Low	Low	Some concerns	Low	Some concerns
Vargas et al.,^23^ 2024	Low	Low	Low	Some concerns	Low	Some concerns
Pedley et al.,^27^ 2018	Low	Low	Low	Some concerns	Low	Some concerns

D1: Randomization process; D2: Deviations from intended interventions; D3: Missing outcome data; D4: Measurement of the outcome; and D5: Selection of the reported result.

## DISCUSSION

The goal of this rapid review was to evaluate the effectiveness of CHW-led interventions as a method of hypertension management among underserved U.S. urban populations. Of 377 original studies, 10 longitudinal studies were assessed on the basis of the inclusion criteria. Although these findings suggest that CHW-led interventions may contribute to improved blood pressure control and self-management behaviors, the strength of this evidence must be interpreted within the context of study design limitations and the hierarchy of evidence. Heterogeneity in intervention design and delivery between studies must also be considered before generalizing the results to other urban environments.

The results presented must be interpreted within the context of the study design. Three of the 10 included studies were RCTs.^21^^,^^23^^,^^27^ Notably, 2 of the 3 RCTs did not demonstrate statistically significant differences in SBP between the intervention and control groups.^23^^,^^27^ Only 1 RCT (Ursua and colleagues^21^) reported a significant 20 mmHg reduction in SBP at 8 months compared with controls. These findings suggest that when accounting for nonspecific effects such as regression to the mean, the evidence for CHW-led interventions improving blood pressure is mixed.

One study was a non-RCT, and 6 studies used quasi-experimental pre–post designs without concurrent control groups.^19^^,^^20^^,^^22^^,^^24^, ^25^, ^26^^,^^28^ Although 4 of these studies reported statistically significant within-group blood pressure reductions, these findings must be interpreted cautiously.^19^^,^^22^^,^^25^^,^^26^ Blood pressure declines are commonly observed even in control arms of hypertension trials owing to regression to the mean, Hawthorne effects, and secular trends. Without a comparison group, it is not possible to attribute the observed changes directly to the CHW intervention.

Of the included studies, 60% reported statistically significant reductions in blood pressure, with systolic reductions ranging from 2.3 to 20 mmHg and diastolic reductions ranging from 1.6 to 7.4 mmHg.^19^^,^^21^^,^^22^^,^^25^^,^^26^^,^^27^ These results are clinically significant, because current evidence indicates that every 5 mmHg decrease in SBP translates to roughly a 10% lower risk of suffering major cardiovascular events and stroke.^29^ Interventions targeting participants with uncontrolled blood pressure were associated with improved hypertension control, suggesting that high-risk patients may benefit from receiving longitudinal support from CHWs.^22^^,^^25^

Several study characteristics played a critical role in the outcome of each intervention. All interventions that were conducted for a period greater than 6 months led to significant reductions in blood pressure, suggesting the importance of intervention length.^21^^,^^27^ The results indicate that interventions prioritizing hypertension management as a primary goal were more successful than those where blood pressure control was a secondary outcome for assessing CHW effectiveness.^19^, ^20^, ^21^, ^22^, ^23^, ^24^, ^25^, ^26^, ^27^, ^28^ Furthermore, hybrid approaches that combined personalized CHW sessions with group-based education were effective, likely because they allowed participants to learn broadly about heart-healthy behaviors while receiving tailored care.^21^^,^^27^ Finally, the analysis reveals that collaborative models in which CHWs worked alongside specialized providers, specifically dieticians or pharmacists, were associated with greater improvements in blood pressure and secondary outcomes.^19^^,^^27^ In view of the conclusions drawn from prior meta-analyses, we propose that sustained, individualized CHW engagement, combined with a group-based educational structure and support from other healthcare professionals, may be an effective strategy to help patients with hypertension to better manage their blood pressure.^14^^,^^15^^,^^16^

Adherence to prescribed antihypertensive medication regimens is an important factor in hypertension management. However, nonadherence in the U.S. is reported to be as high as 50%, which is strongly associated with negative effects on patient outcomes.^5^ An important finding in 2 studies reported in the literature review indicates that CHW support may have led to improved medication adherence for participants, either by directly addressing behavioral changes or by working in collaboration with pharmacists.^19^^,^^22^

Even in studies without blood pressure changes, CHWs facilitated significant behavioral improvements, including increased physical activity and self-monitoring.^20^ These changes occurred during the 3-month study and may have led to significant blood pressure improvements over a longer timeframe, considering that the majority of other successful interventions lasted for at least 6 months.

The greatest change in blood pressure outcomes was observed with a CHW intervention that increased healthcare engagement through better appointment keeping with physicians.^21^ This result, combined with the other changes in self-management behaviors observed, reinforces the notion that CHWs may play an important role in amplifying the effectiveness of traditional medical care by addressing the structural barriers associated with appointment and medication adherence.

Given the evidence presented among the reviewed studies and in consideration of the current literature on CHW-led interventions, we argue that, with further research, there may be a rationale for establishing an insurance reimbursement program for hypertension management in the future, similar to the Diabetes Prevention Program (DPP) developed for patients with diabetes by the Centers for Disease Control and Prevention in 2010.^14^^,^^15^^,^^16^^,^^30^ The DPP has demonstrated both clinical and economic value, with Zhou et al.^31^ reporting that lifestyle interventions following the DPP curriculum achieved a median incremental cost-effectiveness ratio of $6,212 per quality-adjusted life year gained, compared with $13,228 for non-DPP interventions. As such, a structured, evidence-based CHW-led hypertension program modeled on the DPP, with insurance coverage, may expand accessibility and provide longitudinal support to high-risk, medically underserved urban populations, improving their cardiovascular outcomes while generating long-term health system savings.

The primary implication of this review is that CHW-led interventions are associated with improved hypertension outcomes in underserved urban U.S. communities. Future research should continue to explore optimal intervention characteristics to inform the development of a scalable, insurance-reimbursable model similar to the DPP.

### Limitations

This rapid literature review lacked a priori registration. In addition, consistent with rapid review methodology, the authors searched a single database (PubMed), which may have missed eligible studies indexed only in other databases. Despite the authors intentions to maximize generalizability with the study criteria, the conclusions are limited by the small sample size of included interventions and their unstandardized approach in how CHWs were selected and trained, which may confound how effectively they educated and supported study participants with hypertension management. For example, 90% of study participants from Lopez and colleagues^20^ reported high satisfaction with their CHW, even though no significant blood pressure changes were achieved. On the contrary, recordings of CHW sessions from Lynch et al.^22^ revealed poor intervention fidelity with regard to creating action plans for study participants, and yet significant blood pressure changes were observed, and participants highly rated the acceptability and appropriateness of the intervention. Furthermore, the results of multicomponent interventions leveraging a team-based collaborative approach with pharmacists, nurses, or dieticians may have been primarily driven by these other healthcare professionals as opposed to CHWs.^19^^,^^24^^,^^27^

In consideration of the recently updated guidelines from the American College of Cardiology/American Heart Association to treat patients to an SBP <120 mmHg and DBP <80 mmHg in certain patient populations, it is important to acknowledge that the studies reviewed did not utilize these criteria.^32^ The American College of Cardiology/American Heart Association further recommends the use of the new PREVENT risk calculator, which includes quantifying a social deprivation index to capture the social determinants of health that may increase an individual’s absolute 10-year risk for total cardiovascular disease.^33^^,^^34^ These changes, by definition, increase the number of individuals currently living with uncontrolled hypertension, strengthening the urgent need to improve lifestyle interventions, especially for underserved U.S. urban communities that are most vulnerable to poor hypertension management.

The scope of this review was further limited to the U.S. population and a timeframe of studies published between 2015 and 2025 because the inclusion criteria focused on the most recent literature. This review was limited to the PubMed database and excluded other databases, which may contain potentially eligible studies. The authors acknowledge that comprehensive systematic reviews typically search multiple databases. However, the rationale for focusing on PubMed was to complement prior meta-analyses that have systematically searched other major databases. Specifically, Mills and colleagues^14^ searched MEDLINE, Embase, CINAHL, and the Cochrane Central Register of Controlled Trials; Patil et al.^15^ searched MEDLINE, Embase, CINAHL, PsycINFO, and CENTRAL; and the Community Preventive Services Task Force conducted comprehensive multidatabase searches, but this was conducted in 2015.¹⁶ Notably, none of these prior reviews systematically searched PubMed as a standalone database. By focusing exclusively on PubMed, this review provides complementary coverage to the existing evidence base while reducing redundancy. In addition, 1 eligible study was identified through review of reference lists and prior knowledge in the topic area, representing supplementary hand searching. Nevertheless, the authors acknowledge that potentially eligible studies indexed only in databases not searched may have been missed, and future reviews should consider multidatabase approaches to maximize comprehensiveness.

Formal statistical assessment of publication bias was not conducted because heterogeneity precluded meta-analysis, rendering such tests inapplicable. However, several factors suggest that publication bias may affect the evidence base. First, studies demonstrating significant blood pressure reductions may be more likely to be published than those with null findings. Second, this review identified only peer-reviewed, published studies. Third, the predominance of pre–post designs without control groups may reflect a tendency to publish studies showing within-group improvements.

## Appendix Supplementary materials

Image, application 1

Image, application 2
