# Supplementary material for: Evaluating Longitudinal Community Health Worker–Led Interventions for Improving Hypertension Management in Urban U.S. Communities: A Rapid Review
Source: AJPM Focus. 2026 Sep 10;5(6):100527. doi: 10.1016/j.focus.2026.100527 (PMC13586563; doi:10.1016/j.focus.2026.100527)
Supplement: Supplementary file 1 [file mmc1.pdf]

Title: "Evaluating Longitudinal Community Health Worker-Led Interventions for Improving Hypertension Management in Urban US Communities: A Rapid Review"

Authors: "Sagar Rastogi, BS; Nisa Maruthur, MD, MHS; Maxwell Droznin, MD, MPH"

Journal: "AJPM: FOCUS"

Title Complete PubMed (MEDLINE) Search Strategy and Dates

Database and platform

- Database: PubMed (MEDLINE)
- Platform: PubMed (https://pubmed.ncbi.nlm.nih.gov)

Date last searched

- 01/01/2015-05/01/2025

Eligibility time frame and limits

- Publication years: 01/01/2015-05/01/2025
- Language: English
- Species: Humans
- Note: No geographic filter applied in PubMed; U.S. urban setting was applied during screening.

Concept blocks and terms

- Concept 1: Hypertension/Blood Pressure Hypertension[Mesh] OR hypertension[tiab] OR "high blood pressure"[tiab] OR "blood pressure"[tiab]
- Concept 2: Community Health Workers "Community Health Workers"[Mesh] OR "community health worker*"[tiab] OR promotora*[tiab] OR "lay health worker*"[tiab] OR "peer navigator*"[tiab] OR "patient navigator*"[tiab] OR "community health aide*"[tiab] OR "health promoter*"[tiab]
- Concept 3: Urban Setting "Urban Health"[Mesh] OR "Metropolitan Areas"[Mesh] OR urban[tiab] OR "inner city"[tiab] OR city[tiab] OR metropolitan[tiab]

- Concept 4: Intervention/Program intervention*[tiab] OR program*[tiab] OR trial[tiab] OR randomized[tiab] OR randomised[tiab] OR "pre-post"[tiab] OR "before and after"[tiab]

Exact PubMed query to run (Hypertension[Mesh] OR hypertension[tiab] OR "high blood pressure"[tiab] OR "blood pressure"[tiab]) AND ("Community Health Workers"[Mesh] OR "community health worker*"[tiab] OR promotora*[tiab] OR "lay health worker*"[tiab] OR "peer navigator*"[tiab] OR "patient navigator*"[tiab] OR "community health aide*"[tiab] OR "health promoter*"[tiab]) AND ("Urban Health"[Mesh] OR "Metropolitan Areas"[Mesh] OR urban[tiab] OR "inner city"[tiab] OR city[tiab] OR metropolitan[tiab]) AND (intervention*[tiab] OR program*[tiab] OR trial[tiab] OR randomized[tiab] OR randomised[tiab] OR "pre-post"[tiab] OR "before and after"[tiab])

Apply PubMed filters

- Publication dates: Custom range 2015/01/01 to 2025/12/31
- Species: Humans
- Language: English

Record management and screening

- Export all records (e.g., as .nbib or .csv).
- Deduplicate in your reference manager or screening tool.
- Title/abstract screening and full-text review conducted independently by two reviewers; disagreements resolved by discussion.
- Apply inclusion criteria during screening:
    - Urban U.S. setting
    - Participants recruited from community settings (not clinical care settings)
    - Longitudinal CHW involvement
    - Pre- and postintervention blood pressure reported
- Apply exclusion criteria during screening:
    - Rural settings; studies outside the U.S.
    - Recruited from clinical/healthcare settings
    - No pre/post blood pressure outcomes

- Non-original research (reviews, commentaries)
